# Development of an intravenous chemotherapy intervention for children and adolescents with cancer administered by their parents at home (INTACTatHome)

**DOI:** 10.1186/s12913-023-09613-2

**Published:** 2023-06-20

**Authors:** Louise Ingerslev Roug, Martha Krogh Topperzer, Rikke Thenning Michelsen, Mary Jarden, Ayo Wahlberg, Lisa Lyngsie Hjalgrim, Helena Hansson

**Affiliations:** 1 Department of Pediatric and Adolescent Medicine, Copenhagen University Hospital, Rigshospitalet, Copenhagen, Denmark; 2 Faculty of Health and Medical Science, University of Copenhagen, Copenhagen, Denmark; 3 Department of Pediatrics and Adolescent Medicine, Copenhagen University Hospital, Rigshospitalet, Copenhagen, Denmark; 4 Department of Pediatrics and Adolescent Medicine, Copenhagen University Hospital, Rigshospitalet, Copenhagen, Denmark; 5 Department of Hematology, Center for Cancer and Organ Disease, Copenhagen University Hospital, Rigshospitalet, Copenhagen, Denmark; 6 Department of Clinical Medicine, University of Copenhagen, Copenhagen, Denmark; 7 Department of Anthropology, University of Copenhagen, Copenhagen, Denmark; 8 Department of Pediatric and Adolescent Medicine, Copenhagen University Hospital, Rigshospitalet, Copenhagen, Denmark

**Keywords:** Pediatric oncology, Childhood cancer, Parents, Health care professionals, Experiences, Home chemotherapy, Complex intervention, Development

## Abstract

**Background:**

Families of children and adolescents with cancer strive to maintain routines and normalcy during the child’s treatment trajectory that requires frequent hospital visits. Intravenous chemotherapy at home can reduce time spent on the frequent hospital visits and mitigate disruption in daily life. Studies on home chemotherapy for children and adolescents with cancer are limited, as is knowledge of family and health care professionals’ needs, and knowledge required to inform adaptation or replication of interventions in other settings. The aim of this study was to develop and describe an evidence-based home chemotherapy intervention that is feasible and safe for children and adolescents and suitable for future feasibility testing.

**Methods:**

The Medical Research Council’s guidance for developing complex interventions in health care and the framework of action developed by O’Cathain et al. was used as theoretical frameworks to structure the development process. A literature search, an ethnographic study, and interviews with clinical nurse specialists from adult cancer departments formed the evidence base. Educational learning theory to support and understand the intervention was identified. Stakeholder perspectives were explored in workshops with health care professionals and parent-adolescent interviews. Reporting was qualified using the GUIDED checklist.

**Results:**

A stepwise educational program to teach parents how to administer low-dose chemotherapy (Ara-C) to their child at home and a simple and safe administration procedure were developed. Key uncertainties were identified, including barriers and facilitators impacting future testing, evaluation, and implementation. Causal assumptions and reasoning for how the intervention leads to short-term outcomes and long-term impact were clarified in a logic model.

**Conclusions:**

The iterative and flexible framework allowed for integration of existing evidence and new data and was successfully applied to the development process. The detailed report on the development process of the home chemotherapy intervention can enhance adaptation or replication of the intervention to other settings and thereby mitigate family disruption and stress of frequent hospital visits for these treatments. The study has informed the next phase of the research project that aims to test the home chemotherapy intervention in a prospective single-arm feasibility study.

**Trial registration:**

ClinicalTrials.gov ID: NCT05372536.

**Supplementary Information:**

The online version contains supplementary material available at 10.1186/s12913-023-09613-2.

## Background

Families of children diagnosed with cancer strive to maintain routines and normalcy during the child’s treatment trajectory that often requires frequent hospital visits [1–3]. Administration of intravenous chemotherapy at home can reduce time spent at the hospital and mitigate disruption in the everyday lives of the child and families [4–10]. Furthermore, studies suggest that home care with intravenous chemotherapy for children and adolescents is feasible and safe [9, 11–13] and can decrease chemotherapy related side effects, e.g. nausea and vomiting, and reduced well-being [5, 7, 8, 14, 15]. Intravenous chemotherapy at home can be administered by a nurse from the municipality, home care agency or hospital or by the parent or primary caregiver. Studies show that parents are willing to undertake a variety of home care tasks such as medicine administration and Central Venous Catheter (CVC) care to avoid hospitalization and maintain normalcy in their daily lives [7–9, 16–18]. However, Kelly et al. and Stevens et al. found that the complexity of intravenous home chemotherapy can be experienced as a barrier by health care providers and recipients [19–21]. Medication management at home is high-risk and errors are common [22, 23]. Parents can become anxious and insecure, hence needing support to provide complex care for their child at home [6, 17, 24]. To meet the needs of health care professionals, children, adolescents, and their families it is therefore imperative to involve them when developing home chemotherapy interventions.

Studies on home chemotherapy interventions for children and adolescents with cancer are limited. Moreover, there is a diversity in design, procedures, and outcome measures of interventions, reflecting the complexity of home chemotherapy services [8, 13, 25–27]. Only few studies provide the information needed to assess the extent to which home chemotherapy interventions are feasible and suitable for adaptation or replication in other settings. This includes information on an intervention’s development, contextual implications, evaluation, and implementation, including barriers and facilitators [28–30]. Thus, systematic and comprehensive descriptions of development and evaluation of home chemotherapy interventions are needed [30–32]. The aim of this study is to develop and describe an evidence-based home chemotherapy intervention that is feasible and safe for children and adolescents and suitable for future feasibility testing.

## Methods

### Theoretical development framework

Home chemotherapy interventions can be defined as complex due to a number of interlinking components, actions and behaviors required by providers and recipients, targeted groups and organizations, variability of outcomes, and degree of intervention tailoring [29, 33]. The research project INTravenous AntiCancer Treatment for children and adolescents at Home—INTACTatHome (ClinicalTrials.gov ID: NCT05372536) was designed using the Medical Research Council’s (MRC) guidance for developing and evaluating complex interventions in health care [29, 33]. The present study describes the development phase of the home chemotherapy intervention according to the three elements of the MRC guidance (2008): 1) identifying the evidence base; 2) identifying/developing theory; and 3) modelling processes and outcomes [33]. The updated MRC guidance (2021) defines six core elements to be applied to all phases of complex interventions: consider context; develop, refine and (re)test programme theory; engage stakeholders; identify key uncertainties; refine the intervention; and consider economic factors. All of the above-mentioned elements were applied to the development process [29], which was further supported by the framework of actions developed by O’ Cathain et al. [30]. Both the MRC guidance and O’Cathain et al. suggest a dynamic and iterative development process that is open to change and invites evaluation and implementation from the start [29, 30]. O’Cathain et al. emphasize that careful planning and conduct of intervention development can enhance feasibility, effectiveness and implementation in real world clinical practice [30]. User participation was advised to ensure relevance and acceptance of the intervention by the target groups [29, 30]. Therefore, perspectives of health care recipients and providers were included throughout the development process. Reporting was qualified using the GUIDED checklist [31].

### Setting

The intervention was developed at the Department of Pediatric Oncology and Hematology (DPOH) at the Copenhagen University Hospital in Denmark. Approximately 100 patients aged 0–18 are diagnosed yearly at the DPOH. In Denmark, paid leave of absence from work is provided to one parent, financed by the State, during the child’s active treatment [34].

### Intravenous home care service at the DPOH

There is no outgoing nursing service from the DPOH or any established collaboration with community-based home care nurses or home care agencies in providing intravenous treatment for children and adolescents at home. A hospital-based Home Care Unit (HCU) was established at the DPOH in 2018 providing intravenous antibiotic, antifungal, and a few other treatments as home care using portable infusion pumps (PIP) and with no requirements of assistance of a nurse at home. The HCU is an outpatient service located near the inpatient ward and outpatient unit and only include children, who will continue the intravenous treatment they received at the inpatient ward at home. At the HCU, HCU nurses initiate the intravenous therapies using PIPs and provide parents with instructions on how to observe the PIPs and manage different types of alarms at home. Depending on the child’s treatment, HCU nurses can also teach families more advanced procedures such as changing an infusion bag connected to a PIP or connecting and disconnecting a PIP from the child’s CVC. There is close collaboration with home care treatment delivery between the HCU nurses and the staff of the outpatient unit, inpatient ward, and the nurse specialist (RTM). Inquiries from families and patients after daytime hours are managed by the DPOH inpatient staff. At the time of the present study, the HCU did not provide intravenous chemotherapy as home care. Intravenous chemotherapy is provided as either inpatient treatment, whenever overnight hydration and monitoring is needed, or as outpatient treatment at the ambulatory or outpatient unit.

### The intervention development group

An intervention development group was established [30] and comprised first author (LIR), nurse specialist (RTM), senior researcher and clinical nurse specialist (HH), chief nurse and chief clinician (LLH) at the DPOH. The group had decision-making authority at both the clinical practice and organizational levels regarding content, form and delivery of the intervention [30]. Other stakeholders and specialists were included on an ad hoc basis such as pharmacists affiliated at the DPOH and pharmacists working at the hospital pharmacy, experts in the electronic patient journal system, DPOH physicians and DPOH nurse specialists in chemotherapy administration and central venous catheter (CVC) care.

## Development of the intervention

**Fig. 1:**
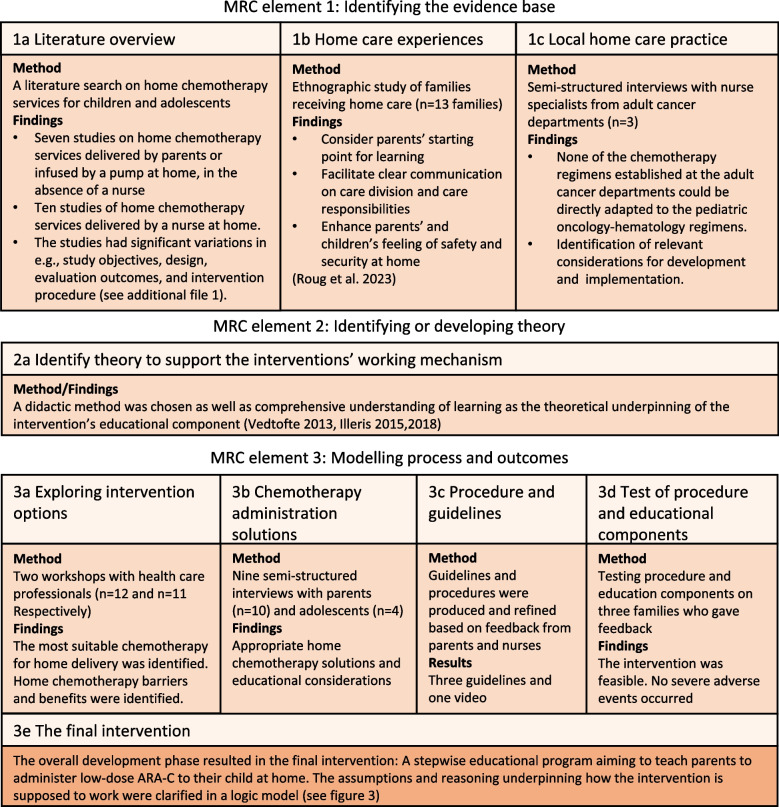
Overall development process of the home chemotherapy intervention

The development process took place from July 2020 to March 2022. Figure 1 illustrates the steps in accordance with the three elements of the MRC guidance (2008) (see Fig. 1).

### MRC element 1: Identifying the evidence base

To inform the evidence base, a literature search on intravenous home chemotherapy services was conducted as well as an ethnographic study on children with cancer and their parents’ experiences of home care, and three interviews with nurse specialists from local adult departments [30, 33].

#### Literature overview

A literature search on intravenous home chemotherapy services for children and adolescents was conducted using the CINAHL and PubMed databases, supplemented by a snowball search strategy. We identified seven studies in which home chemotherapy was delivered by parents or infused by a PIP, in the absence of a nurse [8, 9, 11–14, 25], and 10 studies in which home chemotherapy was delivered by a nurse [5, 7, 10, 14, 15, 27, 35–38] (see Additional file 1). The literature search showed that the studies had significant variations in study design, outcomes, and procedures e.g., whether a single drug or more drugs were offered for home treatment. Few studies elaborated on the development process of the services or on considerations for implementation such as facilitators and barriers [5, 6, 8, 25, 35]. Three studies raised attention to the risk of parents perceiving home chemotherapy as a possible burden [6, 8, 25]. One study reported two reasons why home chemotherapy could not be delivered as intended, despite extensive preparation and pilot testing [25]. The first reason was a lack of outpatient care resources while the second related to clinician reluctance to transfer and oversee treatment in an outpatient setting [25]. The literature search resulted in an overview of intravenous home chemotherapy services and pointed to key uncertainties, barriers, and facilitators to consider in the development process as well as the need for clear objectives for an intervention [29, 30].

#### Home care experiences and needs of families of children with cancer

An ethnographic study was conducted to explore experiences with home care in thirteen families having children with cancer to identify pivotal family needs to consider when developing the intervention [17]. The study showed that parents were willing to manage different home care tasks to avoid hospitalization and that their perspectives on complexity did not always correspond to what health care professionals define as complex e.g., regarding intravenous treatments [17]. The study also demonstrated that families experienced ambiguous expectations of parent caregiving at home and at the hospital from nurses, their children and themselves [17]. Parents’ needs for clear communication on the division of care responsibilities, their need for safety and security while providing care at home, and their individual prerequisites for learning the care tasks were included in the development process. The study is reported in full in a previous publication [17].

#### Relevant home chemotherapy solutions and considerations for implementation

Semi-structured interviews were conducted with three clinical nurse specialists from three adult cancer departments with experience in home chemotherapy services to identify relevant intravenous home chemotherapy solutions, benefits, and barriers. The interviews were recorded and transcribed. One department had administered only 5-FU chemotherapy using an elastomeric infusion pump. The other two departments provided multiple chemotherapies and hydrations as home care using an electronic PIP [39, 40]. Organizational changes imposed on the two departments, i.e., moving inpatient care to home care managed by the outpatient ambulatory unit, significantly influenced care routines. The clinical nurse specialists informed that since their patients were at home more, they had to reorganize care to deliver all required information and instructions within a shorter timeframe. Ward nurses and clinicians appreciated the comprehensive support from the implementing team when adapting to the new routines of prescribing and administering chemotherapy to the home setting as well as education and regular meetings to discuss their concerns. Initially, clinical nurse specialists met resistance from staff but that decreased when the staff saw how the home chemotherapy services benefitted the patients. None of the home chemotherapy regimens established at the adult cancer departments could be directly adapted to pediatric oncology-hematology. Practical and organizational elements that emanated from the interviews as well as experiences with development and implementation were discussed in the intervention development group and informed the development process and future process evaluation outcomes.

### MRC element 2: Identifying or developing theory

A theoretical perspective can help inform content, delivery, evaluation, and the intended goals of the intervention [30, 33]. We chose to focus on theory to underpin the educational part of the intervention.

#### A didactic model and learning theory to support intervention design and evaluation

Moving chemotherapy administration from the hospital into a patient’s home entails new nursing care procedures and educational practices for nurses and the parents. Vedtofte [41] suggests a broad didactic model when planning and evaluating education in health care. The model includes eight essential elements: overall goal, specific purpose, participant prerequisites, relationships between those involved in the educational context, content, methods, practicalities, and evaluation [41]. The model was used to development the intervention’s education program. Illeris presents a comprehensive understanding of learning that comprises four types of learning, perspectives on barriers to learning, and internal and external conditions [42, 43]. The didactic model and the learning theory were used as analytical instruments to understand both recipients and provider learning processes in the development phase and will be used when evaluating the parent and the nurse experiences with teaching and learning in the intervention.

### MRC element 3: Modelling process and outcomes

Modelling the intervention process and outcomes requires prioritizing, selecting, and refining the intervention components by synthesizing knowledge gained during the development phase. New data collection was necessary to specify perspectives, needs and preferences of both providers and recipients and support decision-making [32, 33]. The modelling process was based on five elements: 1) two workshops on suitable home chemotherapies, barriers, and benefits; 2) interviews on specific types of home chemotherapy solutions; 3) procedure and guideline development; 4) testing procedure and educational components; and 5) modelling the final intervention and outcomes.

#### Health care professional perspectives on home chemotherapy interventions

Two workshops were conducted with health care professionals (HCP) comprising focus groups [44] combined with elements from the Nominal Group Technique (NGT) [45–47]. NGT is used to structure an equal consensus building process in a group with different perspectives and controlled by a facilitator [45, 47]. The aim was to explore, discuss and seek consensus on chemotherapy treatments suitable for home chemotherapy in the absence of a nurse, as well as identify potential facilitators and barriers. NGT elements were used, including individual ranking of the chemotherapies, stringently facilitated group discussions that allowed for equal speaking opportunity, and plenum discussion. DPOH nurses and physicians were invited to participate in two workshops. Nine nurses and three physicians attended Workshop 1 (n = 12) while nine nurses and two physicians attended Workshop 2 (n = 11). All participants had pediatric oncology and hematology expertise in chemotherapy management in addition to more than five years of clinical practice at the DPOH. Department management was represented by the chief nurse and chief physician. LIR was the primary facilitator while MKT, RTM, and a research nurse assisted at the workshops. Prior to Workshop 1, the participants received information material on the current home care service at the DPOH to align their knowledge [45]. The material included a list of 13 chemotherapy treatments with low anaphylactic reactions and requiring multiple consecutive infusion days, derived from 211 pediatric cancer protocolss used at the DPOH. The treatments were divided into three categories; short infusions (≤1 hour), longer infusions (>1 hour), and continuous hydration. The participants individually assessed suitability of the specific home chemotherapies by ranking them on a five-point scale before and after the workshop.Each workshop contained nine structured 15-minute focus group sessions, with 4–5 participants in each group. Each participant took part in three focus groups on predetermined themes during a workshop. The participants wrote down ideas, reflections, and conclusions on cardboards during the focus groups. The focus group discussions were recorded and transcribed. Data (transcriptions and cardboard notes) were coded by LIR using NVivo and analyzed deductively to identify suitability of chemotherapy for home treatment including barriers and facilitators. The analysis was subsequently discussed in the intervention development group. Notes from the plenary discussions were included in the data.

##### Workshop 1

Perspectives identified in the focus groups: 1) most suitable chemotherapies for home treatment: Short infusions with low-dose cytosine arabinoside (Ara-C), 24 hours infusion with doxorubicin, and hydration after cyclophosphamide and iphosphamide; 2) barriers to home chemotherapy: Work reorganization, potential initial financial loss, increased educational support as part of care, increased care coordination, and management of severe side effects at home; and 3) benefits of home chemotherapy services: Reduced side effects, more time at home, education as an investment, optimized organization, and potential financial savings.

##### Workshop 2

Nurses and physicians elaborated on topics identified in Workshop 1 as most important for the success of home chemotherapy interventions including: a well-planned educational program; strict coordination of care and treatment; and adequate nurse resources. Sepsis resulting from parental management of the CVC, extravasation of doxorubicin infusion at home on a Port-a-Cath, and bad compliance were identified as the most important risk factors.

The results suggested low dose (Ara-C) as the most suitable objectivechemotherapy for the intervention.Perspectives from both workshops were discussed by the intervention development group and informed the intervention objectives and design, outcome measures, and future implementation.

#### Parent and adolescent perspectives on specific home chemotherapy solutions

The parents (*n* = 10) and adolescents (*n* = 4) were interviewed individually (parents) or in dyads (parent-adolescent) to determine needs and preferences related to specific home chemotherapy solutions and educational requirements. A convenience sampling strategy was used, and interviews were conducted during admission to the DPOH. Participants had experience with intravenous antibiotic/antifungal infusions at home with PIP or hospital care with the chemotherapies assessed relevant for home care intervention during the workshops (see Table 1 for participant characteristics). RTM conducted the interviews using a short semi-structured interview guide (see Table 2) and demonstrated the different types of equipment used in home chemotherapy, e.g., electronic PIP, elastomeric PIP, and syringes for chemotherapy injections, after which participants were asked about their personal experiences with any of the equipment and preferences for home use. Questions on treatment solutions and education moved between reflection on prior experiences and hypothetical considerations about preferences. Oral and written consent was obtained from all participants and the interviews were recorded and transcribed verbatim. Data were analyzed by RTM, MKT and LIR using reflexive thematic analysis [48, 49].

**Table 1:** Participant characteristics in parent-adolescent interviews

Parents	*n* = 10
Fathers	3
Mothers	7
Child/Adolescent	*n* = 9
Present during interview	8
Participating in the interview	4 (aged 14–16)
Sex	
Female	5
Male	4
Age	
Average	9,2 (1—16)
Diagnosis	
Ewing sarcoma	1
Adrenal cancer	1
Osteogenic sarcoma	2
Acute lymphoblastic leukemia	4
Infant acute lymphoblastic leukemia	1
Prior treatment experience	
Intravenous chemotherapy and hydration at the hospital	9
Electronic infusion Pump at home^a^	6
Elastomeric infusion pump at home^a^	2
Days from diagnosis to interview	
Average	182 (41–463)

^a^Supportive medicine such as antibiotic or antifungal medicine

**Table 2:** Interview guide for parent-adolescent interviews

Treatment:
• You have tried to get (example of treatment) at the hospital, how would you consider having this treatment at home?
• How would you feel about that?
• In what way would you prefer having it? Examples depend on what is relevant for the treatment (by an electronic portable infusion pump, an elastomeric infusion pump or by injection)
• Why do you prefer it this way?
• What are the benefits?
• What are your concerns, worries and what obstacles do you see?
• What do you think about taking on home care tasks?
Training/education:
• What kind of education or training would you need to manage the treatment at home?
• Do you have anything to comment on prior experiences of training and education related to chemotherapy, hydration, or PIP treatment? what was good/bad?

#### Findings

**Fig. 2:**
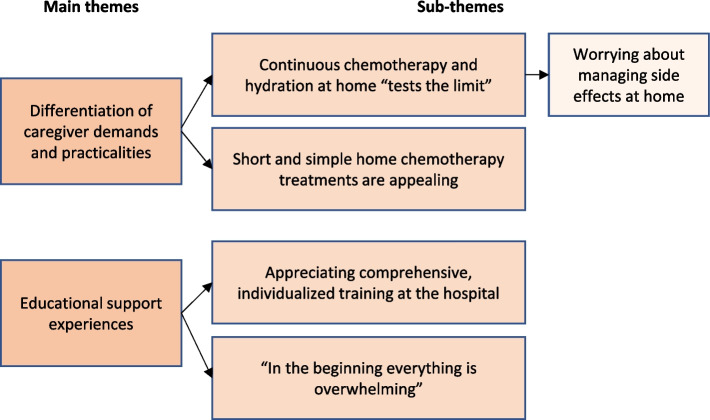
Overview of main and sub-themes identified in the parent-adolescent interviews

Two main themes and fives sub-themes were identified (see Fig. 2). Main themes, sub-themes, and representative quotes are provided in Additional file 2.

#### Main theme 1. Differentiation of caregiver demands and practicalities

*Continuous chemotherapy and hydration at home “tests the limit”.* Most parents and adolescents found that care tasks during continuous chemotherapies and/or hydrations at home would be demanding and burdening. Some considered the idea of continuous intravenous hydration at home as manageable while simultaneously acknowledging the comprehensive caregiver demand it would imply. Others expressed reluctance to assume the tasks and focused on specific issues, e.g. where to rinse out urinals or how to keep track of pH-values:

*” I think there are more challenges to it... because firstly, it's actually a method that requires quite a lot of gadgets (...) we need urinals, we need (...) those pH-sticks... we can easily bring them home..., but then you start accumulating a lot of gear... uhm... I don't know... it could be done, but I think it would be testing our limits...”. (Father to P1, 12 years old).*

Finally, some parents and adolescents found the sum of care tasks to be overwhelming but were unable to offer specific explanations. Both parents and adolescents appeared more reluctant to administer chemotherapy at home compared with hydration, which they generally considered less risky.

*Worrying about managing side effects at home.* Severe and harmful side effects were a constant concern for the parents and adolescents. Similar to findings in the ethnographic study [17], the parents doubted their ability to observe and manage severe treatment related side effects at home during continuous chemotherapy and/or hydration. An adolescent, who had experienced side effects during treatment at the hospital, seemed concerned about whether this would be manageable at home:

*"I don't exactly know why, but when [getting hydration and chemotherapy] I really like being in here [at the hospital] … also because then you get nauseous and [experience] all kinds of pain… I've been in pain and stuff like that… then I think it's very good to be in here" (P9, 16 years old).*

A mother stated that being a single parent is a challenge when managing harmful side effects during home chemotherapy infusions. Another mother underscored that it can be more difficult to assess symptoms and side effects in a young child.

*Short and simple home chemotherapy treatments are appealing.* Parents found solutions with chemotherapy administered by syringe and injected into a CVC or infused through elastomeric PIP appealing due to their simplicity and speed and stressed the feeling of independence and normalcy they could provide. However, administering the chemotherapy by syringe also prompted uncertainty: *“And you don't get sick receiving it in a really short time span, like you do with that one?” (*Mother to P5, 4 years old). One mother reflected on the practical challenges related to administer the chemotherapy by syringe: *“(…) It can, of course, be difficult to sit still, when you’re as young as* [*name of the child*] (…)*”, (Mother to P7, 1 year old).* Parents and adolescents considered the short elastomeric pump infusions and chemotherapy solutions by syringe injected into the CVC as more non-restrictive to daily life than the electronic PIP. However, some parents with elastomeric PIP experience noted that it took some effort to learn the procedure and that they had to keep an eye on the pump to monitor the proper infusion rate and correct position during infusions. None of the participants had experience with injecting medicine directly into the CVC.

#### Main theme 2. Educational support experiences

*Appreciating comprehensive, individualized training at the hospital.* Parents and adolescents who had experienced home care with antibiotics or antifungal treatment on a PIP described their appreciation of comprehensive instructions from the HCU nurses including written guidelines and supervised hands-on training. Both parents and adolescents emphasized hands-on training as a pivotal prerequisite to feeling confident in managing medical home care tasks. The parents trusted the nurse’s assessment and decision of when they were ready and competent to manage the care tasks:

*“(…) Alpha Omega is that you feel secure. They’re very good at informing that they won't send you home until you’re confident that you can manage it and they’re confident that you can.” (Mother to P7, 1 year old)*

However, one mother and her daughter described feeling insecure being supervised during a training session at the hospital while they felt calm and secure practicing the same procedure at home.

*In the beginning everything is overwhelming.* Readiness and timing were seen as pivotal by the parents and adolescents regarding learning and managing medical care tasks at home. While most participants agreed that learning and managing complex care tasks were sometimes introduced too early, there were different views across and within families on when the right time should be. Parents stated that it was important for them to learn and have experience in administering oral medicines and performing basic care tasks before additionally managing new complex care tasks at home:

*“I can understand if someone feels insecure about managing some of those things themselves, but we just haven't felt that way. I'm sure we can learn it [home treatment].” (Father to P8, 2 years old). But it also depends on when in the process you’re introduced to it… [because] at the start everything is overwhelming … you have to learn so much about the tube and stuff like that, but um... later.” (Mother to P8, 2 years old)*

In conclusion, the findings showed that parents and adolescents assessed shorter infusions by means of an elastomeric PIP and by injection into CVCs to be manageable, simple, and fast while continuous infusion of hydration and chemotherapy were considered more complex and demanding. For some, the benefits of being at home outweighed the added workload of continuous hydration and chemotherapy, while others remained reluctant to use this solution. There is a need for comprehensive and individualized educational support and hands-on training, the timing of which should be carefully considered by the staff. Findings were discussed in the intervention development group and subsequently incorporated in the intervention development process and evaluation outcome measures.

#### Procedure and guideline development

Evidence-based procedures and guidelines were developed in parallel with and following the workshops and parent-adolescent interviews, and included: 1) non-touch CVC care procedures using a needle-free connector; 2) adaptation of local guidelines for chemotherapy induced nausea and vomiting control in home chemotherapy; 3) procedure for administering Ara-C by injection instead of infusion; 4) delivery procedure of Ara-C from the hospital pharmacy, 5) guidelines for prescribing and documenting parent-administered chemotherapy at home in the electronic patient journal; 6) a nursing guideline on how to teach the parents, ensuring the fidelity of the intervention; 7) a parent guideline on how to perform the procedure providing step-by-step instructios and safety precautions.  A demonstration video showing the procedure was also produced.

#### Testing the home chemotherapy procedure and educational components

We tested the intervention with low-dose Ara-C administered by parents at home to assess safety, educational components, adaptation to clinical care practice, and integration with the electronic patient journal system. Three families were invited to participate using a purposeful sampling strategy. Two of the families were recruited prior to Ara-C treatment and one family was recruited after cycle two of Ara-C. The parents were instructed in critical reading of all information and guidelines. The first child received 11 of 16 doses Ara-C at home with no complications. The second child received 17 of 24 doses of Ara-C at home with five minor complications, and the third child received 14 of 16 doses of Ara-C at home with one minor complication. No severe adverse events occurred during the testing. The types of minor complications were: 1) nurses forgot to inform parents to flush the CVC between treatments; 2) a booking error caused delivery of infusion bags instead of syringes by the pharmacy; 3) incorrect connection of the safety caps on the chemotherapy syringes delivered by the pharmacy; 4) parents forgot to close the CVC tap before disconnecting the injection syringe; 5) nurses forgot to install the needle-free connector before discharging the patient. Adjustments were made and feedback were given to the relevant stakeholders e.g., pharmacists delivering the syringes with cytarabine. Close collaboration with the hospital pharmacy was imperative to quickly adjust preparation and delivery of chemotherapy to align with the requirements of the home care organization. The procedure guidelines and parent information materials were adjusted according to suggestion of the parents. The procedure for home chemotherapy prescription and documentation were refined by the electronic patient journal specialist who reviewed all subsequent prescriptions to ensure their accuracy. Testing the intervention’s educational components revealed that flexibility in delivery time and place was imperative to meet the individual needs of the parents. The test also showed that the families managed more doses of chemotherapy at home than expected.

#### Final intervention and outcome modelling

The intervention development group modelled the final intervention based on the results from all development process components, acknowledging that further refinements may be needed when testing the feasibility [30].

##### Logic model

**Fig. 3:**
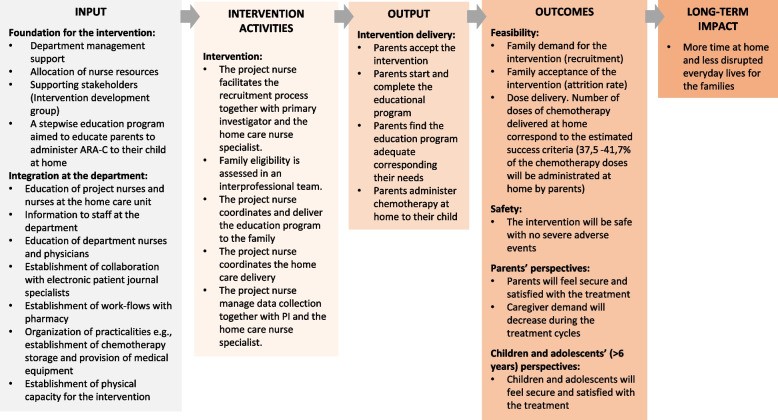
Logic model of the home chemotherapy intervention

A logic model was created to forecast expectations of the intervention in clinical practice [50]. The logic model aims to clarify the causal assumptions and reasoning underpinning how the intervention is supposed to lead to short-term outcomes and long-term impact [32]. The logic model included five basic components: 1) inputs and resources to deliver the intervention; 2) intervention activities; 3) outputs as immediate results of the intervention; 4) outcomes related to feasibility, safety, satisfaction and caregiver demands; and 5) long term impact [51, 52] (see Fig. 3). The logic model was discussed in the intervention development group and refined throughout the development process.

##### Intervention description

The intervention consisted of a stepwise educational program aiming to teach parents to administer low-dose Ara-C to their child at home. The intervention could also be entrusted and managed by an adult caregiver close to the child or adolescent other than the parents. Eligibility criteria for children and adolescents: Ara-C in the treatment protocol and a medical condition that meets DPOH standards before discharge. Exclusion criteria for parents: Inadequate caregiver resources and inability to read and understand guidelines in Danish or English. No restrictions based on proximity to the hospital. The stepwise educational program followed this progression: Step 1) Approximately 0–14 days before the chemotherapy starts, the nurse informs the parents and the child/adolescent about the home chemotherapy intervention. If the family needs time to consider their participation, a follow-up meeting will be scheduled. The family may start or decline the education program whenever they choose. If the parents consent, the nurse then delivers detailed information on the procedure and introduces the guidelines, an instruction video, and a practice kit with medical equipment, as well as demonstrating the procedure on a mannequin torso equipped with a CVC. Step 2) The parents practice the procedure at the hospital or at home. The nurse supervises the parents practicing the procedure and provides feedback. The parents practice until they manage the procedure independently. Step 3) The parents flush the CVC with a dose of sterile saline water to their child, as if it was the chemotherapy, supervised by a nurse. Then they proceed to administer the first dose of Ara-C. The first dose is always administered at the DPOH. When the parents feel secure administering the chemotherapy, and the nurse assesses that the procedure is being managed correctly, the additional information on observation and adverse event management is provided and explained to the parents. Only then do the parents proceed with chemotherapy administration at home in the consecutive days. The chemotherapy procedure takes 10—15 min, including unpacking and clean-up of medicine and equipment. A follow-up call from the nurse is mandatory after the first chemotherapy administered at home. The nurse documents the parents’ education and training process in the electronic patient journal.

## Discussion

The study aimed to develop an evidence-based home chemotherapy intervention that was safe and suitable for future feasibility testing, and to report the development process comprehensively and transparently. MRC guidance for developing complex interventions in health care and O’Cathain et al.’s framework of actions [29, 30] were successful in supporting the systematic development of the complex parent-led home-administered low-dose Ara-C intervention for children and adolescents with cancer. Furthermore, the iterative and flexible frameworks allowed integrating existing evidence with new data. The comprehensive and transparent description of the development process can enhance adaptation or replicability in other settings [28, 30–32].

Stakeholder involvement in development of complex interventions is imperative to achieve intended changes and to ensure maximum impact [29, 30]. However, purpose and manner of involvement must be carefully considered [29, 30]. McCall et al. is the only study identified in our literature search that mentions involvement of parents in the development process to ascertain a demand for a home chemotherapy program [8]. Our study included both provider and recipient perspectives in the development phase. However, pitfalls for stakeholder involvement in research processes are acknowledged, as addressed by Malterud and Elvbakken (2020) who state that stakeholder involvement does not always add new knowledge and can even compromise scientific quality [53]. In the present study knowledge was revealed and clarified on parents’ willingness to administer chemotherapy injections, their significant preference for and appreciation of structured hands-on training, as well as the importance of considering parents’ readiness prior to the intervention.

Low-dose Ara-C was identified as the most suitable parent-led home-administration chemotherapy. Three other studies have reported similar interventions with Ara-C as parent-administered home chemotherapy [8, 9, 14]. Inclusion criteria in the studies were parents’ competences and experiences with the CVC, in e.g, flushing with saline, administering heparin or in blood sampling. The testing of the procedure and the educational components in the present study showed that all three participating families learned to manage the CVC, regardless of having had prior expertise. As such, we argue that previous experience with the CVC should not be an inclusion criterion. The three studies recommended extensive education prior to the home chemotherapy intervention in which this study complies [8, 9, 14]. Although all studies emphasize the importance of comprehensive education and training, and acknowledge the influence on the clinical care practice for nurses, they do not elaborate on the impact of the same [8, 9, 14]. Changes in clinical care practice for intravenous home chemotherapy interventions in the current study are identified by the workshop HCPs as barriers in two main topics: 1) “Increased educational support as part of care”, and 2) “Increased care coordination”, indicating potential key uncertainties to be cognizant of when testing feasibility of the intervention and as relevant evaluation outcome measures [29–31]. Martins et al. identify “care coordination” as one of three core elements of the critical worker’s care process in a comprehensive study that explores and defines the role of the nurse specialist in UK [54, 55]. Although care coordination in these studies is explored within a context whereby shared care centers are well-established in the pediatric cancer care, it underlines the family need for care coordination and elucidates some of the challenges to be attentive to when complex care is transferred to the home.

Due to the different amounts of Ara-C cycles in the protocols, feasibility outcomes on exact use of the interventions are not easily compared. More specific evidence of feasibility in terms of demand, acceptance, dose and practical coordination of treatment delivery is needed [56]. When implementing home administration of chemotherapy agents to larger groups of pediatric oncology patients, it is vital to continuously align expectations with parents and involve them in the process. This ensures that parents are aware of their responsibilities and allows for adjustments in caregiver tasks based on changing needs and available care resources. To provide home administration of chemotherapy as part of standard care services, competent and experienced nurses are needed. To ensure adherence to protocols, the establishment and coordination of home chemotherapy should always be carried out through interprofessional collaboration with clinicians, nurses, pharmacists, and other experts. Moreover, it is important to prioritize stringent documentation of the treatments administered at home.

Avoiding complications as those occurring in the intervention test reported in this study must be considered before undertaking further feasibility testing or guiding other groups wishing to implement home chemotherapy [30]. As the complications in this study related to nursing communication to families, family misunderstandings, and pharmacy errors in drug delivery, communication becomes a key element. In this intervention communication is strengthened by the theoretical framework of the education program. To ensure safe communication, fidelity of delivering the education program to parents as intended should be documented as seen in the study by McCall et al. [8]. Before testing home chemotherapy interventions on families, it is crucial to address and take into consideration high risk adverse events such as accidental swapping of intravenous Ara-C syringes with intrathecal Ara-C syringes or spilling chemotherapy. Precautionary measures should be taken to minimize these risks to ensure safety and wellbeing of the children and families.

### Strengths and limitations

The comprehensive explorative approach of this study, including new data strengthens the development of the study and encourages feasibility in clinical practice. The dynamics in the parent-adolescent interviews show that parental and adolescent perspectives are not always aligned but represent different viewpoints. In some cases, the adolescents expressed disagreement with what their parents considered as problematic in the intravenous home care treatment. In other cases, the parent misjudged the adolescent’s appreciation of being at home. A limitation of the interview study is that only five adolescents and no younger children were included in the data collection. Thus, the voice of adolescents and children are underrepresented. No known study on pediatric intravenous home chemotherapy in the absence of a nurse, that we know of, has included the adolescent or child’s perspective. Another limitation to the present study is that the data collected only represents a single pediatric cancer department.

## Conclusion

This study delivers a detailed report on the development process of a home chemotherapy intervention that used iterative and flexible frameworks allowing for integration of existing evidence and new data. Stakeholder involvement throughout the development process ensured that families’ and health care professionals’ perspectives and needs were included and addressed. Low-dose Ara-C was identified as the most suitable home chemotherapy for the intervention, based on stakeholder ranking and discussion. A safe procedure for parent-administrated low-dose Ara-C was developed with a stepwise educational program, inspired by recommendations of corresponding interventions. The detailed report on the development process can enhance adaptation or replication of the intervention to other settings to mitigate family disruption and stress of frequent hospital visits for these treatments. The study has informed the next phase of the research project that aims to test the home chemotherapy intervention in a prospective single-arm feasibility study.

## Supplementary Information

**Additional file 1.** Overview of studieson home chemotherapy interventions for children and adolescents with cancer. 

**Additional file 2.** Parent-adolescent interviews. Main themes,sub-themes, and representative quotes.

## Data Availability

All data generated or analyzed during this study are included in this published article and its additional files (Additional files 1 and 2). For more information on intervention guidelines please contact the corresponding author.

## Abbreviations

MRC: The Medical Research Council
HCP: Health care professional
DPOH: Department of Pediatric Oncology and Hematology at Copenhagen University Hospital, Rigshospitalet in Denmark
CVC: Central venous catheters
HCU: Home care unit
PIP: Portable infusion pump
DNVK: National Committee on Health Research Ethics
