# Supplementary material for: Development of an intravenous chemotherapy intervention for children and adolescents with cancer administered by their parents at home (INTACTatHome)
Source: BMC Health Serv Res. 2023 Jun 20;23:664. doi: 10.1186/s12913-023-09613-2 (PMC10283184; doi:10.1186/s12913-023-09613-2)
Supplement: Supplementary file 1 — Additional file 1. Overview of studieson home chemotherapy interventions for children and adolescents with cancer. [file 12913_2023_9613_MOESM1_ESM.docx]

| **Additional file 1.** Overview of studies on home chemotherapy interventions for children and adolescents with cancer |
| --- |
| Studies on home chemotherapy services delivered by parents or infused by a pump at home, *in the absence of a nurse:* |
| 1. McCall, C. et al. *Administration of Home Intravenous Chemotherapy to Children by their Parents: Parents’ Evaluation of a Nationwide Program.* 2017 |
| 1. Hooker, L. and Kohler, J. *Safety, efficacy, and acceptability of home intravenous therapy administered by parents of pediatric oncology patients.* 1999 |
| 1. Zelcer, S. et al. *The Memorial Sloan Kettering Cancer Center experience with outpatient administration of high dose methotrexate with leucovorin rescue.* 2008 |
| 1. Mahadeo, K.M. et al. *Ambulatory high-dose methotrexate administration among pediatric osteosarcoma patients in an urban, underserved setting is feasible, safe, and cost-effective.* 2010 |
| 1. Lashlee, M. and O’Hanlon Curry, J. *Pediatric home chemotherapy: Infusing “quality of life.”* 2007 |
| 1. Jayabose, S. et al. *Home chemotherapy for children with cancer.* 1992 (Intravenous chemotherapy administered as injection) |
| 1. Kelly, K.P.et al. *Caregiving demands and well-being in parents of children treated with outpatient or inpatient methotrexate infusion: A report from the children’s oncology group.* 2014 |
| Studies of home chemotherapy services delivered *by a nurse at home:* |
| 1. Ranney, L. et al. *Letting Kids Be Kids: A Quality Improvement Project to Deliver Supportive Care at Home After High-Dose Methotrexate in Pediatric Patients With Acute Lymphoblastic Leukemia.* 2020 |
| 1. Lippert, M. et al. *The Hospital at Home program: no place like home.* 2017 |
| 1. Bartholomew, J.L. Et al. *Feasibility of Outpatient High-Dose Methotrexate Infusions in Pediatric Patients With B-Lineage Acute Lymphoblastic Leukemia.* 2018 |
| 1. De Zen, L. et al. *Safety and Feasibility of Home Transfusions in Pediatric Palliative Care: A Preliminary Report.* 2022 |
| 1. Close, P. et al. *A Prospective, Controlled Evaluation of Home Chemotherapy for Children With Cancer*. 1995 |
| 1. Lange, B.J. Et al. *Home care involving methotrexate infusions for children with acute lymphoblastic leukemia.* 1988 |
| 1. Stevens, B. et al. *Hospital and home chemotherapy for children with leukemia: A randomized cross-over study.* 2006 |
| 1. Hansson, H. et al. *Hospital‐based home care for children with cancer: Feasibility and psychosocial impact on children and their families.* 2013 |
| 1. Kok, N.T.M. et al. *Chemotherapy intravenously in children with cancer at home, the nurse practitioner makes it possible.* 2019 |
| 1. Jayabose, S. et al. *Home chemotherapy for children with cancer.* 1992 (Intravenous chemotherapy administered as infusion) |
