# Supplementary material for: Development of an intravenous chemotherapy intervention for children and adolescents with cancer administered by their parents at home (INTACTatHome)
Source: BMC Health Serv Res. 2023 Jun 20;23:664. doi: 10.1186/s12913-023-09613-2 (PMC10283184; doi:10.1186/s12913-023-09613-2)
Supplement: Supplementary file 2 — Additional file 2. Parent-adolescent interviews. Main themes,sub-themes, and representative quotes. [file 12913_2023_9613_MOESM2_ESM.docx]

| **Additional file 2.** Parent-adolescent interviews. Main themes, sub-themes, and representative quotes. | | |
| --- | --- | --- |
| **Main themes** | **Sub-themes** | **Representative quotes** |
| **1. Differentiation of caregiver demands and practicalities** | 1.1 Continuous chemotherapy and hydration at home “tests the limit” | "I think there are more challenges to it... because firstly, it's actually a method that requires quite a lot of gadgets (...) we need urine bottles, we need (...) those pH-sticks... we can easily bring them home..., but then there starts to be a lot of gear associated with it... uhm... I don't know... it could be done, but I think it would be testing the limit...”, father, P1, 12 years old.  "But the whole hydration show, I think it's actually nice that there are professionals to do the monitoring. Yes, I don't think I really wanted to do that.”, mother, P5, 4 years old.  "But it is also more the practicality of it (…) it will almost be a bigger show to go back and forth only to have diuretics, (...) it will actually be worse to go back and forth all those times”, mother, P5, 4 years old.  "I can't quite figure that out, because right now, for example, we are washing the urine bottles in the special washing machine, which will not be available at home... so what is the alternative? Are there disposable urine bottles? or do we have to wash them ourselves? and how should they be washed up, for example....”, Mother, P3, 14 years old.  "So, it's starting to get quite comprehensive... and um... I think... first... then it's probably something that would be, what should we say... for... the cancer super user, that is, um... those who sort of have, the necessary… competence and experience in doing it”, father, P1, 12 years old.  ”So, I think it's necessary to be here (at the hospital)... for chemotherapy and most of the treatment... but it's also okay when I have those times with fluids... when it's not a really big project to hold an eye on... then I think it's perfectly fine, we could learn to do it ourselves... and come home, just for a while (...) just to eat or sleep there... or whatever... and then just come back in … I think that's a good idea”, P6, 15 years old.  "Can you imagine that you had to give some fluid at home?", Interviewer.”  I can easily imagine that. Weighing nappies and diapers at home is a bit more burdensome, but we do it here too. Yes, here we have help at night, but we have not accepted this because (name of the child) wakes up when a nurse comes in and changes. When it's just us changing, he can continue to sleep. It would be perfectly fine to be at home”, mother, P7, 1 year old.  "I think it's a good idea, especially chemotherapies where it's not hugely complex, and there are constantly different things that have to happen..., but where it's more the same, then it seems obvious", father, P9, 16 years old. |
|  | 1.1 Continuous chemotherapy and hydration at home “tests the limit” (cont.) | "HDM, I don't want that at home", P9.  "What would you be worried about here?", Interviewer.  "Well, everything I guess (...), P9, 16 years old.  "But it worked fine, and we were only here because they needed to change the fluid and take blood samples once a day. And it was so taxing just to be here for that little thing and lug around that rack. It was so nice that we got the opportunity to come home for arch, it was boring to be here just for that. Because one thing is to be here when he's ill or receiving treatment, but when he's completely flying, it's boring", mother, P7,1 year old. |
|  | 1.1.1. Worrying about managing side effects at home | "Overall, I think there are some cool things about being able to stay at home... we like that, especially when it comes to some chemotherapy drugs that, like ours, are not as heavy on side effects... if it had been Vincristine or Doxorubicin or some other insanely harsh thing like that, maybe it is nice to be here (at the hospital) so quick side effect relief is possible if needed… and a doctor and things like that”, father, P1, 12 years old.  "But when it's so many hours I wouldn't feel safe but that's because the chemotherapy is so harsh ... it's really harsh.”, mother, P2, 4 years old.  "For me there is a hurdle now... because I have to experience that he... recovers well, after this round of chemotherapy as well... and then it could well be that I would be more open or dare to believe it. The staff also know now that (name of the child) must have maximum anti-nausea medication, and he didn't get that to begin with... so we have had to find out how he reacts... and what works and what doesn't work... um ... yes,... so now we know... it must not be attempted, it must not be graduated like that... it must be given from the start”, mother, P3, 14 years old.  "When I'm alone with (the child), I'd be a bit worried about it... and I must say also with this kind of harsh chemotherapies... um, if it was a slightly milder form, I think I would probably be able to”, mother, P6, 15 years old.  "I think there is a difference in how much they (the children) can cooperate and express things. It's something we still juggle a bit, when is he nauseous, when is he in pain... the older children can express that they feel bad, and they are easier to observe. We have become much better at reading him and predicting, for example, fever and other symptoms", mother, P7, 1 year old.  "I don't exactly know why, but then (getting hydration and chemotherapy), I really like being in here (at the hospital), also because then you get nausea and all kinds of pain…, I've been in pain and stuff like that. Then I think it's very good to be in here", P9, 16 years old. |
|  | 1.1.1 Worrying about managing side effects at home (cont.) | "It could work. We are not concerned with the practical aspect of it (home chemotherapy). What you could be worried about is more (...) Methotrexate or something that has similar side effects..., and where you must hydrate for a very long time afterwards”, mother, P5, 4 years old.  "At that time (during hydration), we came in here (at the hospital) every day anyway, but… it made me feel safe to be sent home… knowing that… there is someone who has just… looked at her, and assessed that she is okay”, mother, P2, 4 years old.  "A prerequisite is that side effects are gone through before discharge, it is not enough with the booklet we have been given, but it is important that it is gone through, so that you know what to react to and what is completely normal”, mother, P7, 1 year old. |
|  | 1.2 Short and simple home chemotherapy treatments are appealing | “(We) clearly prefer that one (solution with syringe), because it is the fastest and the best in her everyday life, the easiest for us, I think, and there is nothing heavy to carry”, mother, P5, 4 years old.  "We would be able to administer it in the morning, for example, before she had to do anything else. When you're sick, normalcy is what you long for, so administer it, we'd dare.”, mother, P5, 4 years old.  "I would dare to. I think that it is something that should be given a little slowly, so you must have a feel for how long it should actually be administered over. It can of course be difficult to sit still when you are as young as (name of the child). Maybe you could have an extension tube (on the CVC) so that you didn't have to stand so close to (the child) but could stand a little at a distance so that he could sit in his chair, for example, so that he doesn't have to lie down or be held if the administration time is 5 -10 minutes.”, mother, P7, 1 year old.  "The last solution is to deliver the chemotherapy undiluted, in four syringes, then you will be taught how to connect the syringe to the diaphragm plug and give the medicine", interviewer.  "Will it take half an hour too?", Mom.  "It will take a few minutes. It's not mixed into anything", interviewer.  "Sounds smart", Mom.  "Yes, then we're done with fixing it (with dressing)", father, P8, 2 years.  "It's even smarter. It is "easy". It doesn't take up that much space either. Are there any downsides to it? At first glance, it seems relatively straightforward as the best solution”, father, P9, 16 years old.  "Can you just inject undiluted chemo?”, P9, 16 years old.  "It can of course be difficult to sit still, when you are as little as (name of the child)”, mother, P7, 1 year old. |
|  | 1.2 Short and simple home chemotherapy treatments are appealing (cont.) | "And you don't get sick getting it over a really short time, like you do with that one? (Referring to the chemotherapy the daughter is having now)", mother, P5, 4 years old.  "Is there anything that... could worry you about this model (elastomeric pump)?”, interviewer.  "Not at all...", P1.  "It doesn't run out of battery. It doesn't alarm in the middle of the night... it's over within a very specific time... which means that somehow, you're freer... Then there's a bit of a hassle when you connect and disconnect it, what I don’t consider especially troublesome... In all I see it is a total win-win, very easy and fast compared to the one that has to run for a longer period of time”, father, P1, 12 years old.  "Cytosar (chemotherapy) given on the easy pump is an advantage because then he can move around. It's easy to put on once you've tried it a few times”, mother, P7, 1 year old.  "I think it works..., well, the downside is that you have to keep an eye on whether it is running properly and... remember to clean it off properly and such, but then... you learn that. Yes, we like it”, P4, 14 years old.  "Yes... but if you didn't know the pump (the elastomeric one) then you might choose the syringe, I think... I don't think I'd take the other pump (the CADD pump)", P4.  "Yes, I'm also thinking about the pump (elastomeric)... I just think it's so easy with that pump, or else I think I would have chosen the other one as well (the syringe)", mother.  "So, it's because you know this one (the elastomeric pump), that you feel it's safe?", interviewer.  "Yes, I think so actually... ", mother, P4, 14 years old.  "Then you don't have to lug around that heavy pump and be connected to it all the time, which is just annoying, even when you go to sleep. I think it is very smart (the elastomeric pump)", father.  "Is there anything you would be worried about with the elastomeric pump?", interviewer.  "Not at all, I think it looks very fun. Then you can control it yourself", P9, 16 years old.  "But it may well be an "issue", (...) for someone getting room in the fridge", father.  “I think the benefits are far greater than that problem (...) Yes, I'd much rather save a few avocados, four days a week and then making room for a few pumps", P9, 16 years old. |
| **2. Experiences of educational support** | 2.1 Appreciating comprehensive and individualized training at the hospital | "I see it as a relatively short process, where you enter the day hospital, for example... and then you sit down, and then they do it... maybe you come at the day hospital the first two days until you feel comfortable with the procedure, and then you say okay now it works, it's fine. But until you are, like, safe, they can, like..., put it on the first time and say: then you do this and remember this and this (...)”, father, P1, 12 years old.  "I was worried that a lot of different nurses would be involved in the training, but it has been nice to see that things can actually be done in different ways and to see more aspects of it”, mother, P7, 1 year old.  "I will be very aware of having to be trained in all that about cleaning properly and keeping it sterile and things like that, because of her recent infection in the CVC and all that. So, I guess I would be completely hysterical about it, but I think we could manage the task”, mother, P5, 4 years old.  "It has been really nice to have some rehearsals in here with the remedies where (name of the child) has not been there, where there has been complete calm and where it has only been us and a nurse. And we've tried to connect the tubes a few times, both of us. The more hands-on training you have, the more comfortable you become with it”, mother, P7, 1 year old.  " (…) Alpha Omega is that you feel safe. They are very good at saying that we won't send you home until you are confident that you can manage it, and we are confident that you will. It is super nice that the staff express that someone needs to be told many times, and some need fewer times (...). It's good that you don't just get sent home. You don't feel pressured to do it, you are allowed to feel for yourself, now I'm actually comfortable with it”, mother, P7, 1 year old.  "We were handed some papers we could read through alongside the instructions we got from the nurse. It was a nice set up on how to do just like this 1, 2, 3, 4, which we could take home to practice. It worked well. Read it through first and then get the instructions from the nurse afterwards, then you could ask any questions, and then we were well prepared. Subsequently, I did a test that the nurse supervised”, father, P8, 2 years old.  "(…), or at least there must be the right education, it may well be that you also have to screen people a bit and say "you are not suitable for that"... you may not need to tell them that, but …”, father, P1, 12 years old.  "It is about getting some "hands-on-training", so you are not coming home thinking, oh, I haven't tried this before. So, you need to get it done a few times in here (at the hospital), and then just do it in here, so that you are not suddenly at home with too little experience?”, father, P9, 16 years old. |
|  | 2.1 Appreciating comprehensive and individualized training at the hospital (cont.) | "And yet, you're actually a little more secure when you're at home... here (at the hospital) you're completely... wow... when someone is watching. At least, that's what (name of the child) and I felt. When we were sitting at home, we were quite good at it, although we might not feel it at the hospital. Yes, there is something about being home in a safe environment”, mother, P4, 15 years old. |
|  | 2.2 “In the beginning everything is overwhelming” | "As soon as we got the opportunity, we could handle it. At that time, we knew what it meant to have a child with cancer, to be in the hospital and to be at home. I think it is important that you have tried to be at home and give medicine in the tube and have the feeling that (the child) is not going to die, just because you are at home. So that everything else you must do at home, has become routine before you can manage more care tasks”, mother, P7, 1 year old.  "I can understand if someone feels insecure about managing some of those things themselves, but we just haven't felt that way. I'm sure we can learn it (home treatment)”, father.  "But it also depends on when in the process you are introduced to it, because in the beginning everything is overwhelming, you have to learn all that with the tube and stuff like that, but um... later”, mother, P8, 2 years old.  "(name of the child) received her first Cytosar (chemotherapy) on day 38, what if you had to be introduced at that time?" interviewer.  "It would have been really nice. I just remember we were in here (at the hospital) all the time (...) to get Cytosar (chemotherapy) or something. It would have been nice to be home. So, I think we would have accepted home treatment at that time. It was also at that time we learned to use the portable infusion pump for antibiotics at home connecting and disconnecting it and shifting infusion bag ourselves.”, mother, P8, 2 years old.  "I really think I could have done it then (home treatment). Yes, (...) what frustrated me the most at that time was that we had to drive in here all the time, a long drive. We didn't spend much time in here, but when we had to drive for half an hour, I thought that was annoying”, P9, 16 years old.  "It's true that you're in a different place at that time (in the beginning of the treatment trajectory), (...) , thoughts are flying around in your head compared to now, when you have a different overview and sharpness that you just didn't have at the beginning, where it's all a bit messy”, father, P9, 16 years old.  "I could imagine that for families who may have been in this for a shorter time than we have… it could be…, I simply think that there are many who wouldn't dare..., but that doesn't mean that it's a bad idea... it's just a question of you have to be in the right place in the process, I think”, father, P1, 12 years old.  "...And I know that we won't get out of that door if they (the nurses and doctors) don't feel safe about it. We have learned that over time", mother, P7, 1 year old. |
